# The Permanent Maintenance of the Voluntary Hospital

**Published:** 1920-01-17

**Authors:** 


④The Hospital

The Workers' Newspaper of Administrative Medicine and Institutional Life, Administration, National Insurance and Health.

No. 1754, Vol. LXVII. SATURDAY, JANUARY 17, 1920. PRICE TWOPENCE

### THE PERMANENT MAINTENANCE OF THE VOLUNTARY HOSPITAL.

On the 23rd inst. Viscount Sandhurst, the Lord Chamberlain, is to preside at probably the most important meeting of the members of the British Hospitals' Association which has been held since its foundation at the Mansion House by Sir Henry Burdett in 1884.

For nearly two months we have been expounding, with some detail and suggestion, the financial difficulties and their solution in which the conclusion of the Great War has placed the voluntary hospitals of Great Britain. Our efforts to help and cheer the managers, who have to bear the heavy responsibility of financing the voluntary hospital in these anxious times, have been reciprocated warmly and heartily, by an increasing number of those who have the permanent maintenance of the voluntary hospital at heart. Our aim and wish have been to create a feeling of enterprise and hope in the breast of everyone concerned, by setting forth the actual position to be dealt with, including the material fact that less than one in ten of the population, at present, practically gives anything to the support of these institutions. A feeling of encouragement has arisen throughout England and Wales, and, as we demonstrated last week by extracts from the great Scotch newspaper, the *Glasgow Herald*, Scotland is becoming alert and awakening to a feeling of readiness to take action, and secure the adequate financing of the voluntary hospitals, there also with practical unanimity. Nowhere has any opposition or adverse voice asserted itself, and in fact the whole country and people of all denominations and interests are becoming fully alive to the importance of maintaining and strengthening the voluntary hospitals in our midst

The hopes which have thus arisen have received substantial encouragement from the results which have attended the spirited efforts of hospital managers in Nottinghamshire, in Bedfordshire, and the North Country, where Warrington Infirmary has led the way. They have further been strengthened and encouraged by the anonymous gifts of several sums of £20,000 each, including one to Guy's Hospital, and one each to Liverpool and Manchester, together with another splendid gift of £50,000 to the Leeds General Infirmary. Our information shows that following the publication in the *Times* of December 17 Sir Henry Burdett's plan to place the voluntary hospitals throughout Great Britain on a sound financial basis (see The Hospital, December 20, page 268), 200 at least of the principal hospitals have awakened to the importance of these proposals and their practicability. Also since the publication of this scheme in the *Times* Sir Henry Burdett has been approached by influential people, who leave no doubt in his mind that, with the combined help, both pecuniary and in personal service, which has already been brought to his notice, the financial crisis, which threatened the voluntary hospitals in Great Britain at the end of 1919, should be adjusted and placed in a fair way to be met and overcome by June 30 next. In this connection we would direct the careful attention of our readers to the statement of Sir Arthur Stanley, the head of the British Red Cross organisation (see page 372) in reference to a scheme which will be fully expounded by him, if all be well, at the meeting of the British Hospitals' Association to be held on the 23rd inst. The road to success points through united forces and the co-operation of a small army of willing workers of both sexes throughout the United Kingdom, so that the canvass of non-givers to our hospitals, amounting in the gross to 90 per cent., and put roughly at 50 per cent. of effective givers of the population, may be possible. Then the claims of the hospitals and the strengthening influence on the individual of the practice of voluntary service in the days of health in the cause of the sick, may be brought home universally, with, we are confident, results, which may astonish and please our readers and the country generally.

Between now and the meeting on the 23rd we should appreciate a line of interest and sympathy from the representatives of the 250 voluntary hospitals to whom we addressed a letter of explanation of the proposed effort to finance the voluntary hospitals and place them on a sound financial basis. In our issue of December 20, page 269, we gave the first results of the information and answers which we received from hospital chairman throughout Great Britain. The chairman and other hospital workers and supporters who are in sympathy with this movement can render material service to-day if each of them will send the Editor a word of cheer, with offers of co-operation whole-heartedly and continuously to June 30 next. Their encouragement will prove the crowning co-operation in the support of the voluntary system of hospitals. This it has never before been possible to organise and set on foot in Great Britain as it can be to-day, since hospitals were first established.

The chairmen of most of the voluntary hospitals of any importance, which are well administered and up to date, have already become members of the British Hospitals' Association (London: 1 Central Buildings, Tothill Street, Westminster, S.W.). It might be well if the chairman of every voluntary hospital who is not already a member were to take the present opportunity to send his name for election to the Honorary Secretary, Mr. J. Courtney Buchanan. The meeting at St. Thomas's Hospital on the 23rd inst. should be attended by the chairman of every voluntary hospital with 100 beds and over, for action is likely to initiate a new era for all interested in and associated with this type of hospital, which is of paramount value to the whole population in Great Britain. Eleven years ago we expressed the hope that the British Hospitals Association and the American Hospitals' Association would join hands, that in due course an Anglo-American Hospital Congress might be organised, to meet with advantage biennially, and alternately, in London and New York, or some other great American city. (See Burdett's "Hospital and Charities," 1909, pages 129-30.)

